# Paramagnetic Agents for SE DNP: Synthesis and ESR Characterization of New Lipophilic Derivatives of Finland Trityl

**DOI:** 10.3390/molecules30224463

**Published:** 2025-11-19

**Authors:** Victor M. Tormyshev, Danil A. Kuznetsov, Arthur E. Raizvikh, Olga Yu. Rogozhnikova, Tatiana I. Troitskaya, Elena G. Bagryanskaya

**Affiliations:** 1 N.N. Vorozhtsov Novosibirsk Institute of Organic Chemistry, Siberian Branch of the Russian Academy of the Sciences (SB RAS), Novosibirsk 630090, Russia; kuznecov@nioch.nsc.ru (D.A.K.); arturaiz@nioch.nsc.ru (A.E.R.); rogol@nioch.nsc.ru (O.Y.R.);; 2 Physical Department, Novosibirsk State University, Novosibirsk 630090, Russia

**Keywords:** trityl radicals, EPR, ESEEM, liposomes

## Abstract

Triarylmethyl radicals (TAMs) have recently emerged as highly effective polarizing agents in dynamic nuclear polarization (DNP) under viscous conditions, enabling substantial hyperpolarization via the solid-effect (SE) DNP mechanism even at room temperature. A comparable, though less pronounced, enhancement was observed for BDPA radicals embedded in phosphocholine-based lipid bilayers. Given the increasing interest in elucidating the structure and dynamics of biopolymers and their high-molecular-weight assemblies—such as cell membranes—this study focuses on the design, synthesis, and characterization of paramagnetic agents tailored for DNP-based structural biology. To this end, we synthesized a series of TAM derivatives functionalized with lipophilic substituents and characterized their magnetic resonance properties, including isotropic hyperfine interaction (HFI) constants on carbon nuclei and electron spin relaxation times (T_1_ and T_m_) at low temperatures (80 K). Echo-detected EPR spectra and electron spin echo envelope modulations (ESEEM) were recorded for novel TAM incorporated into liposomes composed of 1,2-dioleoyl-sn-glycero-3-phosphocholine (DOPC). These low-temperature measurements revealed that the radicals are localized either at the liposome surface or within the lipid bilayer, ensuring optimal accessibility to water molecules. Crucially, the presence of a single cholesterol moiety provides strong noncovalent anchoring within the hydrophobic core of the bilayer. Guided by these findings, we identify an amphiphilic TAM bearing a single cholesterol group and polar carboxyl functionalities as a highly promising candidate for DNP applications in membrane biology, combining efficient polarization transfer, bilayer integration, and aqueous accessibility.

## 1. Introduction

Magnetic resonance imaging (MRI) and in vivo NMR spectroscopy provide unique information crucial for disease diagnosis, treatment selection, and therapeutic monitoring. However, the inherently low sensitivity of NMR, particularly for nuclei such as carbon, phosphorus, and nitrogen, often necessitates long signal acquisition times. This limitation restricts classical in vivo NMR spectroscopy to studying fixed tissue states, making the investigation of dynamic biological processes challenging with conventional techniques. To address this sensitivity barrier, various methods for achieving hyperpolarization of nuclear spins have been developed. Among these, Dynamic Nuclear Polarization (DNP) has emerged as a highly promising tool across diverse fields, including medicine [1,2,3], cellular and tissue studies [4,5,6], biopolymer analysis [7,8,9], and materials science [10,11,12]. While the potential of DNP has been clearly demonstrated for small molecules, typically at low magnetic fields, its application to real biological systems has been primarily confined to solid-state [13] and dissolution DNP [14,15].

Room-temperature DNP was initially realized in 1,2-dioleoyl-*sn*-glycero-3-phosphocholine (DOPC) lipid bilayers using the TEMPOL nitroxide radical at 9.4 T, yielding a 10-fold enhancement via the Overhauser effect [16]. A significant drawback, however, is the broad EPR spectrum of nitroxides in these systems, which makes them difficult to saturate. It was therefore reasoned that paramagnetic agents with narrow EPR lines, such as 1,3-bis(diphenylene)-2-phenylallyl (BDPA) or trityl radicals, should provide a substantial improvement for DNP at ambient temperature [17,18,19,20].

**Figure 1:**
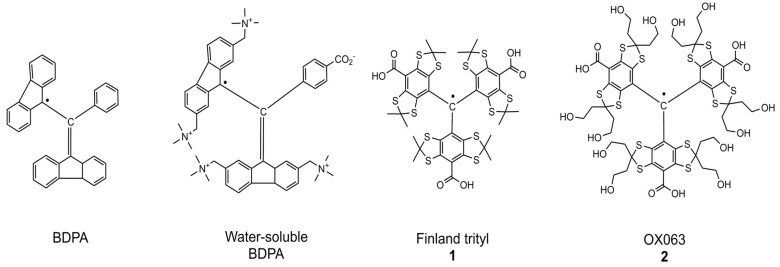
Structures of narrow line organic radicals: BDPA, water soluble BDPA, Finland trityl (**1**), and OX063 (**2**).

Experimental confirmation was provided by a high-field EPR study of 1,2-dimyristoyl-*sn*-glycero-3-phosphocholine (DMPC) lipid bilayers at approximately 320 K, utilizing BDPA (Figure 1) as the paramagnetic agent [17]. Consistent with predictions, BDPA displayed a narrow (5 MHz) EPR signal and produced a substantial DNP enhancement. Contrary to expectations, the resulting DNP spectrum for the lipid protons was antisymmetric, unequivocally identifying the Solid Effect (SE) as the underlying mechanism. Note, however, that the authors of this study, with some caution, named their discovery as “Solid-Like DNP” [17]. The manifestation of the SE at high temperature points to a specific sample characteristic: the synergistic effect of regular lipid ordering and slow molecular diffusion, which endows the system with properties analogous to the solid state, facilitating the normally forbidden excitations.

Given that hindered diffusion at room temperature is typical of high-viscosity media such as glycerol, it was hypothesized that the “Solid-Like DNP” effects observed in lipid bilayers could be replicated in such environments. This was confirmed by studying glycerol solutions containing narrow-line polarizing agents: a water-soluble BDPA derivative and two trityl radicals (Finland (1) and OX063 (2), Figure 1) [18]. At 9.4 T and 315 K, these systems yielded substantial Solid Effect DNP enhancement exceeding 50 for proton nuclei. Notably, signal enhancement of up to 55 was also registered for several tripeptides, underscoring the potential applicability of this approach to biological systems [18].

Subsequent research successfully extended the SE DNP approach to carbon-13 nuclei in viscous media [19]. This was achieved by first generating ^1^H hyperpolarization via the Solid Effect, followed by transferring this polarization to ^13^C nuclei using the INEPT (Insensitive Nuclei Enhanced by Polarization Transfer) sequence. In a model system using glycerol-^13^C_3_ as the analyte and Finland trityl as the polarizing agent, a ^13^C signal enhancement factor of up to 45 was achieved at 9.4 T and ambient temperature. This result, alongside a recent demonstration of ^31^P DNP enhancement exceeding 20 for biomolecules in aqueous solution under similar conditions [20], confirms that substantial hyperpolarization of various nuclei is feasible at high magnetic fields and near-physiological temperatures. These highly encouraging findings underscore the potential for developing high-field SE DNP, particularly through the strategic design of optimized trityl-based polarizing agents.

Currently, the relationship between the structure of narrow-line radicals and their DNP efficiency remains poorly understood. A notable trend shows the ^1^H SE DNP enhancement increasing with the molecular weight of the polarizing agent: water-soluble BDPA (ε = 26) < Finland (ε = 35) < OX063 (ε = 45) [18]. A similar dependence on analyte size was also observed. This correlation has been reasonably attributed to the effect of molecular weight on molecular motion. Heavier molecules experience greater hindrance to their rotation, leading to increased hydrodynamic volume and longer rotational correlation time (τ_c_). This, in turn, enhances the anisotropic hyperfine interactions responsible for the Solid Effect. Expanding on this, one must also consider how the size and rigidity of substituents on the trityl core influence its moments of inertia, as this can introduce significant anisotropy into its rotational diffusion, further modulating DNP performance.

**Figure 2:**
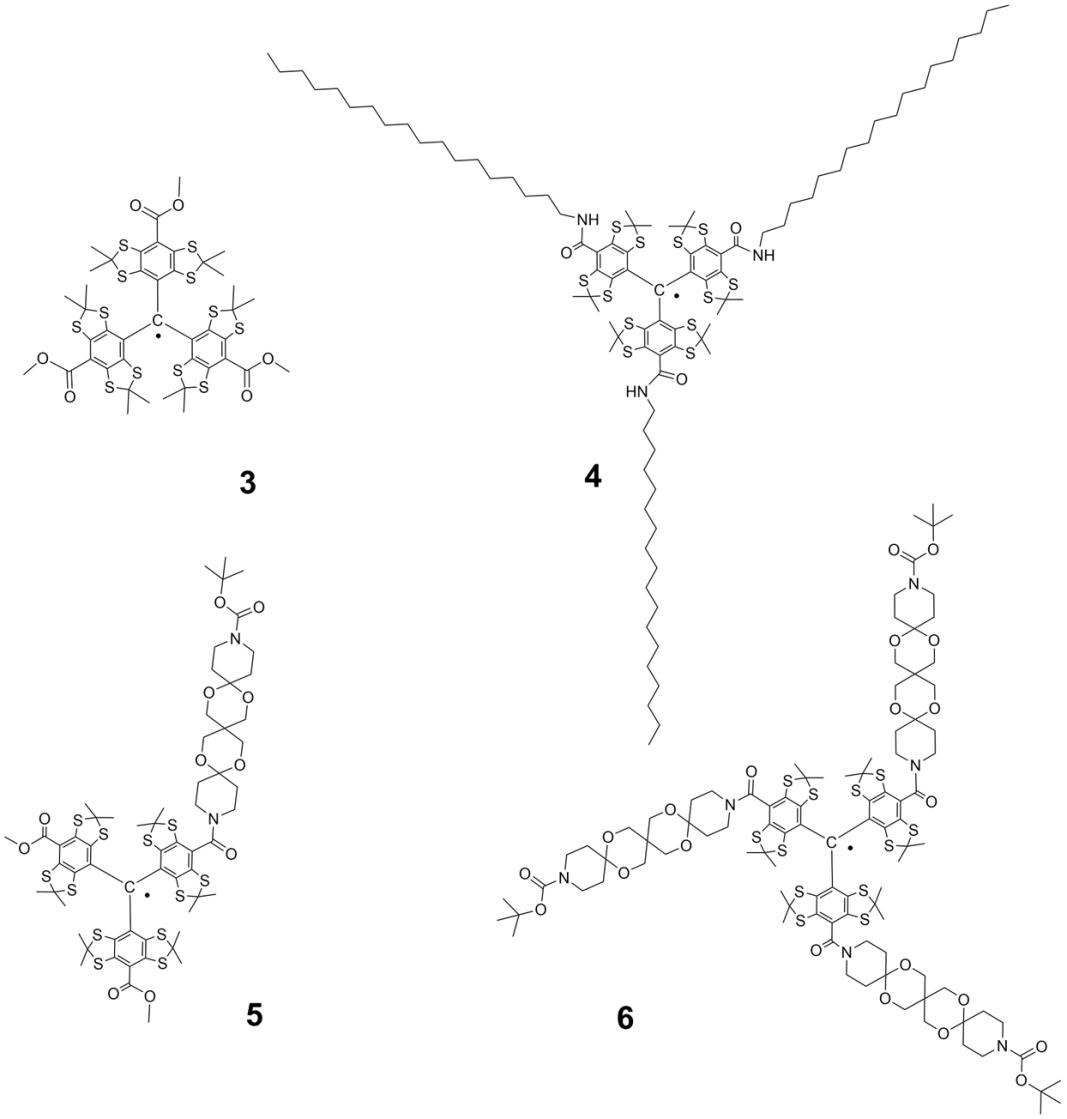
Lipophilic ester- and amide derivatives **3**–**6** of Finland TAM.

**Figure 3:**
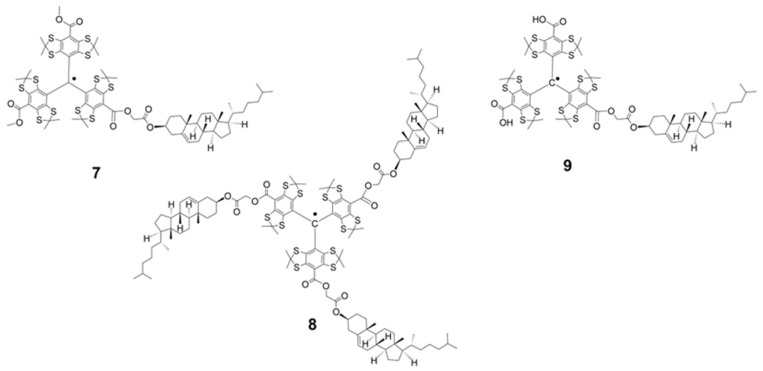
Cholesterol-substituted derivatives of Finland trityl: lipophilic TAMs **7** and **8** and amphiphilic TAM **9**.

This study targeted the synthesis of a new series of Finland trityl (TAM) derivatives (**3**–**9**, Figure 2 and Figure 3) and a systematic investigation of their EPR characteristics to identify advanced polarizing agents. Our molecular design was focused on variation in the hydrodynamic volume, geometric parameters, and rigidity of lipophilic substituents. It is worth noting that the achievement of these goals was facilitated by the rapid progress made in recent years in the field of synthesis and subsequent chemical transformations of triarylmethyl radicals [21,22,23,24,25,26,27,28,29,30]. We obtained mono- and trisubstituted TAMs soluble in low-polarity organic solvents, alongside an amphiphilic derivative (TAM **9**) equipped with a cholesterol anchor and a polar carboxyl group for enhanced versatility. For all compounds (**3**–**9**), we measured critical magnetic resonance parameters, specifically electron spin relaxation times (T_1_, T_2_) and hyperfine interactions. For compound **9**, we studied aggregation behavior with DOPC liposomes via CW-EPR and ESEEM spectroscopy to link molecular structure to membrane localization and solvent accessibility of the radical’s unpaired electron.

## 2. Results and Discussion

### 2.1. Synthesis of the Trityl Radicals

**Scheme 1:**
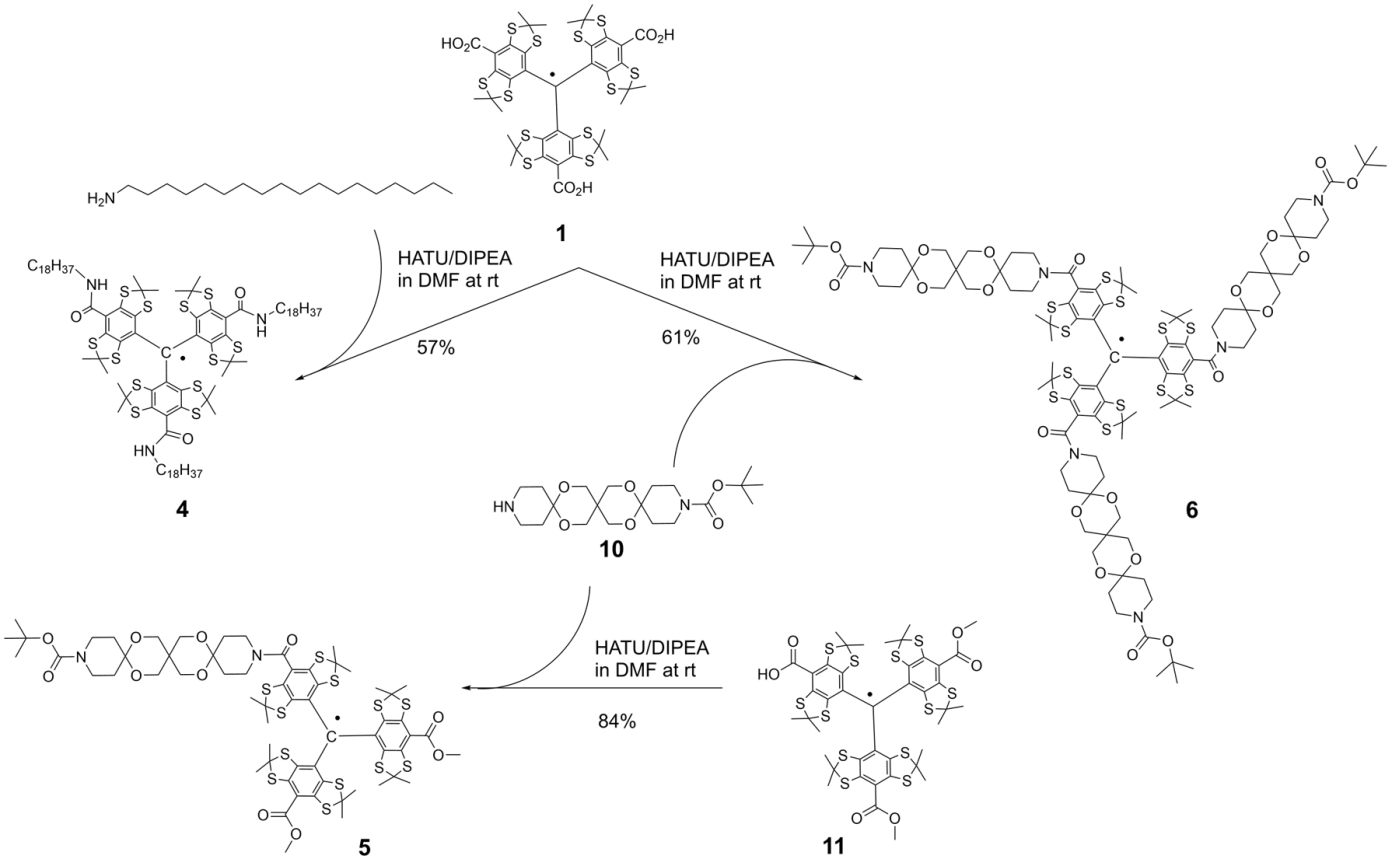
Syntheses of trityl radicals **4**–**6**.

We synthesized trityl radicals **3**–**9** using established methods for Finland TAM functionalization. The trimethyl ester **3** was prepared according to a known procedure [31]. Amide derivatives **4**–**6** were synthesized using modified literature protocols [32,33,34]. As a representative example, TAM **4** was prepared by activating TAM **1** with HATU/DIPEA and subsequent reaction with n-octadecylamine (Scheme 1; see Supporting Information).

The symmetric trityl **6** was subsequently obtained using the same method, with trispiro derivative **10** serving as the monofunctional secondary amine (Scheme 1, Supporting Information). The monosubstituted analog, TAM **5**, was synthesized by coupling the activated form of monobasic acid **11** [33] with amine **10**.

Our initial synthetic strategy for cholesterol-functionalized TAMs **7**–**9**, which involved the direct acylation of cholesterol with TAM acids **1** and **11**, proved ineffective and resulted in poor conversion. We therefore adopted an alternative pathway based on a literature protocol [34]. This method first converts the low-reactivity cholesterol into a more reactive bromoacetic acid ester. The high nucleofugality of the bromine atom then allows for a quantitative esterification reaction with the carboxylic acid functionalities of **1** and **11**, yielding the desired products **7**–**9** efficiently.

**Scheme 2:**
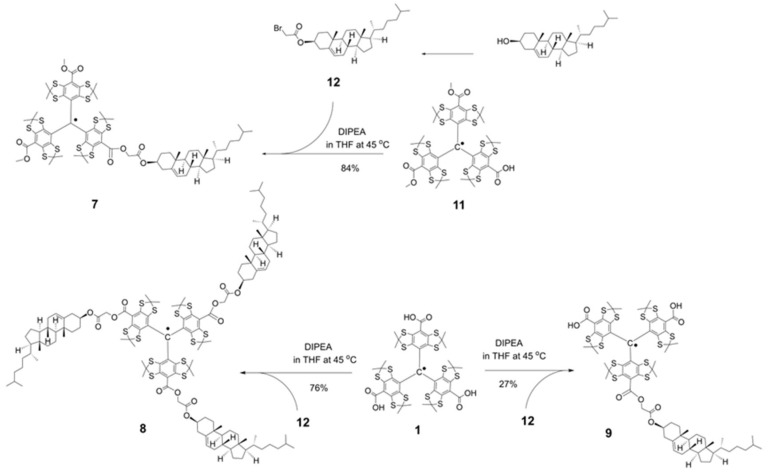
Syntheses of cholesterol-substituted derivatives of Finland trityl: lipophilic TAMs **7**, **8**, and amphiphilic TAM **9**.

Using this alternative route, TAMs **7** and **8** were obtained in near-quantitative yield from the reaction of an excess of bromoacetate **12** with the anionic forms of acids **1** and **11**, respectively (Scheme 2, Supporting Information). Similarly, TAM **9** was synthesized analogously by reacting acid **1** with an equimolar amount of ester **12**.

### 2.2. CW-EPR Studies of the Trityl Radicals

The effectiveness of trityl radicals as polarizing agents in the Solid Effect (SE) DNP mechanism depends critically on two factors: (1) maintaining a narrow, homogeneous EPR linewidth, which reflects a delocalized and unperturbed spin density, and (2) the ability to modulate their dynamics and supramolecular behavior in complex environments such as lipid bilayers. To disentangle these contributions, we first evaluated the intrinsic magnetic properties of the new TAMs (**3**–**8**) in a homogeneous, deoxygenated toluene solution.

**Figure 4:**
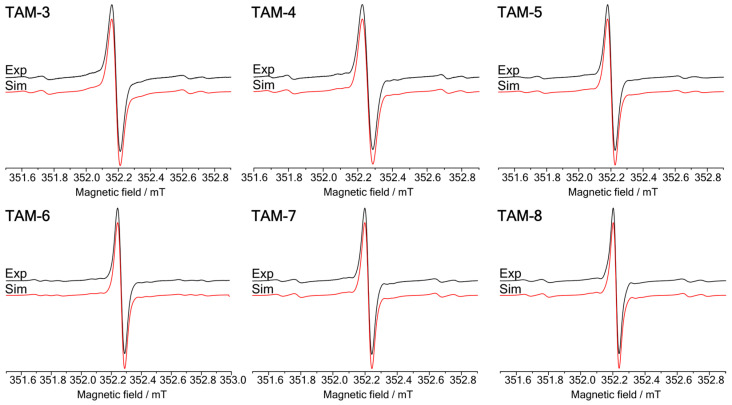
A comparison of experimental (black) and simulated (red) X-band CW EPR spectra of the TAMs **3**–**8** in toluene at room temperature. The TAMs **3**–**8** simulations were performed in the fast-motion regime (fast tumbling of the molecule) with seven spectral components (one ^12^C_1_-TAM and six ^13^C-TAM). An additional ^13^C spectral component was provided during the TAM **6** simulation. ^13^C_1_-TAMs satellites were not included in the presented simulations due to low intensity at the current spectrometer parameters and graph scale. For simplicity of the calculations, the Gaussian and Lorentzian line widths and g-factor values of each ^13^C spectral component were set equal during TAMs **3**–**8** simulations.

The continuous-wave (CW) EPR spectra acquired at room temperature (Figure 4) display the characteristic pattern of ^13^C satellite lines, indicating a molecular symmetry close to C_3_ for the trityl core. Simulations of these spectra using the EasySpin fast-motion model confirmed that the ^13^C hyperfine coupling constants (HFI) are virtually identical for all derivatives (Table 1 and Table 2), varying within a root mean square deviation (RMSD) of ±0.02 mT, in agreement with prior literature [35]. This consistency unequivocally demonstrates that the electronic structure of the trityl core remains unperturbed by the introduction of ester, amide, or cholesteryl substituents, regardless of their number or steric bulk. Consequently, the narrow EPR linewidth—a prerequisite for efficient microwave saturation in SE DNP—is an intrinsic property preserved across the entire series.

Although the electronic structure is conserved, the peripheral substituents modulate intermolecular interactions in solution, as evidenced by changes in the EPR line shapes. The Gaussian contribution to the linewidth (Table 2) scales with the magnitude of unresolved anisotropic hyperfine interactions, consistent with theoretical expectations.

**Figure 5:**
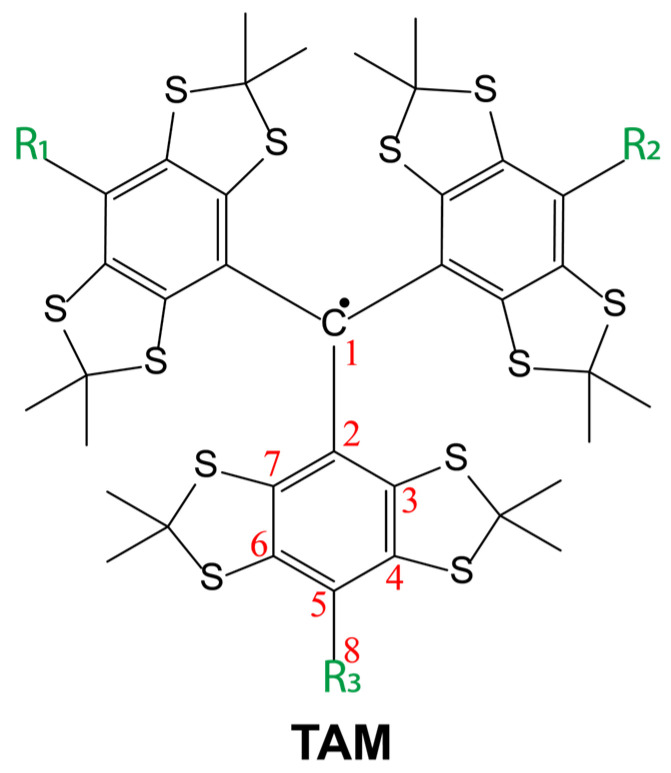
A principal skeletal structure of the TAM radicals (TAM), which represents the 13C carbon atoms with non-zero HFI constant values.

**Figure 6:**
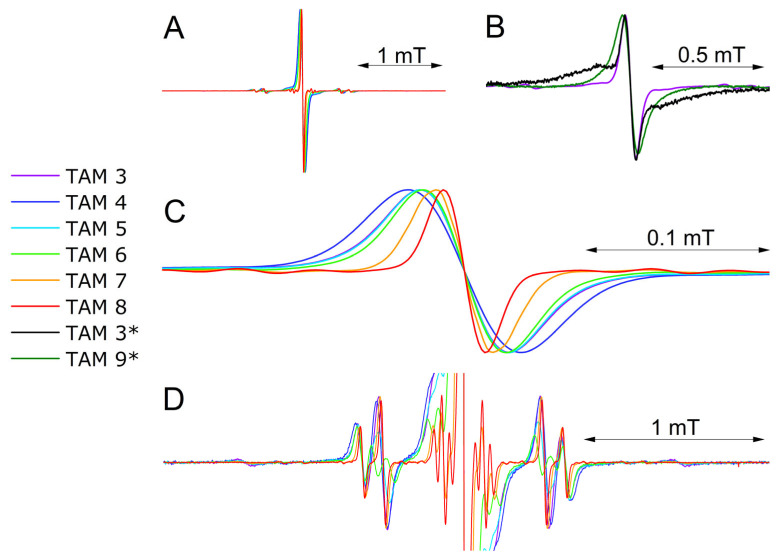
A comparison of the experimental X-band CW-EPR spectra of TAM **3–8** in toluene solution at room temperature: (**A**) full spectra; (**B**) a comparison of the experimental X-band CW-EPR spectrum of TAM **3** in toluene solution at room temperature (purple solid line) and (*) the experimental X-band CW-EPR spectra of TAM **3**, **9** with liposomes at room temperature (black and olive solid lines); (**C**) central ^12^C_1_ line; (**D**) full spectra with rescaled intensity. All presented spectra are placed on the same scale for better comparison.

For all TAM radicals (**3**–**8**), the EPR spectra exhibited the characteristic ^13^C satellite lines with an intensity ratio of 1:3:6:3:6:3:3:6:3:6:3:1. This pattern corresponds to the number of magnetically equivalent carbon atoms in the TAM structure: 1 (C1), 3 (C2), 6 (C3,7), 3 (C5), 6 (C4,6), and 3 (C8) (Figure 5 and Figure 6). The equivalence of carbon pairs (C3/C7 and C4/C6) is a result of fast conformational averaging.

As expected, the EPR linewidth increases with the isotropic hyperfine coupling (HFI) constant, since the primary contribution to line broadening arises from the modulation of the HFI anisotropy, which scales with the isotropic value. The measured ^13^C HFI constants and corresponding linewidths for the ^12^C radicals are listed in Table 1 (with atomic numbering per Figure 5).

Critically, the ^13^C HFI constants are nearly identical for all TAMs studied and match those of the Finland radical. This consistency confirms that the electronic structure of the trityl core is conserved, as previously demonstrated for variations in the number of ester groups, the presence of bulky substituents, or different solvents [35].

A notable exception was observed for an amide derivative with bulky piperidine groups. In this case, hindered rotation around the amide bond breaks the symmetry, rendering the C3 and C7 carbons nonequivalent (Figure 5). This results in a distinct splitting in the EPR spectrum, with two separate ^13^C HFI constants of 0.97 and 0.82 mT (Table 1).

A comparison of the EPR spectra for TAM **3** in toluene and in liposomes revealed a composite line shape in the liposomal environment, consisting of a narrow component, indicative of freely tumbling radicals, and a broad component, which we attribute to radical aggregates on the liposome surface. This broad spectral feature is consistent with previous observations of the Finland TAM in native lipid bilayers [38]. In contrast, TAM **9** is insoluble in toluene but incorporates into liposomes, exhibiting a single, broad EPR singlet. Its linewidth is comparable to the aggregated component of TAM **3** (Table 2), confirming that the amphiphilic TAM **9** is localized within the liposomal membrane.

### 2.3. Pulse EPR, ED-EPR, and ESEEM Studies of the Trityl Radical

To determine the localization of TAM **9** within the lipid membrane, we measured its electron spin relaxation times and performed Electron Spin Echo Envelope Modulation (ESEEM) spectroscopy. At low temperatures (80 K), the electron spin relaxation times of trityl radicals are governed by interactions with surrounding nuclear spins [39,40,41,42,43,44,45]. Consequently, these times are expected to be longer in a deuterated solvent than in a proton-rich environment such as liposomes. We therefore compared the relaxation times of TAM **9** in deuterated methanol and in liposomes suspended in D_2_O (see Supporting Materials Figures S5–S9, Table S1). The kinetics of the electron spin echo decay obtained in the three-pulse ESEEM experiment for TAM 9 with liposomes and D_2_O were successfully simulated by a mono-exponential curve with T_1_ = 0.3 ms, but it fitted better to a stretched exponential curve with T_1_ = 0.14 ms, β = 0.52. ESEEM electron spin echo decay without liposomes in D_2_O or glycerol mixture could not be approximated by a mono-exponential curve and was nicely simulated by a stretched exponential curve with T_1_ = 0.84 ms, β = 0.65.

Electron spin-lattice relaxation T_1_ for trityl radicals has been the subject of many papers, and it was shown that T_1_ for trityl is influenced by temperature, concentration, and the presence of other radicals [32,40,44,45]. In frozen solutions, in the case of high trityl concentration (typical for DNP experiments), relaxation is dominated by spin diffusion to fast-relaxing centers, particularly triads of trityl radicals [39]. At low temperatures, the relaxation rate can also depend on the molecular structure, solution dynamics, and specific experimental conditions such as microwave frequency. It was shown that at temperatures T = 80, the main mechanism is strongly determined by radical concentration [39]. The parameters of T_1_ for 0.2 mM Finland trityl radicals in 1:1 water-glycerol at 100 K were measured by Eaton et al. [31] (e.g., 1.06 ms at 100 K).

The results show a significant decrease in both electron spin relaxation times due to the presence of lipids: the phase memory time (T_m_) decreased approximately twofold (from 4.6 ± 0.2 µs to 2.5 ± 0.2 µs), and the spin-lattice relaxation time (T_1_) decreased threefold (from 0.84 ± 0.1 ms to 0.3 ± 0.1 ms).

The pronounced shortening of T_m_ can be confidently attributed to the increased local concentration of protons from the lipid membrane surrounding the incorporated trityl radicals. The explanation for the T_1_ shortening, however, is more complex and may involve additional factors such as increased local concentration of the trityl radicals themselves and spin-spin interactions within aggregates. Further research, including concentration-dependent measurements, is required to elucidate the precise mechanism for T_1_ relaxation.

ESEEM measurements were performed using a standard three-pulse sequence [46]. In this sequence, two time-separated microwave pulses are followed by a third pulse after an interval, T, after which the stimulated echo is detected. The echo envelope, recorded as a function of T, is modulated by the transition frequencies of nuclei coupled to the electron spin. A key advantage of the three-pulse experiment over the two-pulse method is that it suppresses the sum and difference frequencies of the primary nuclear transitions, thereby simplifying spectral analysis [47,48].

**Figure 7:**
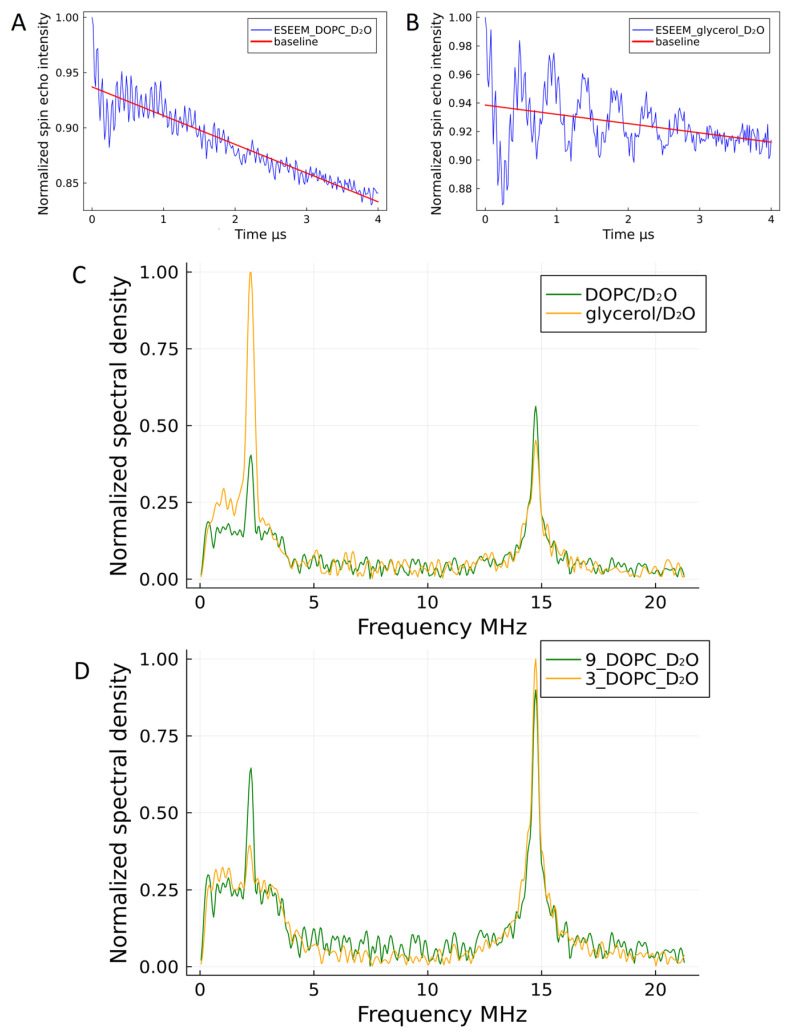
Three-pulse ESEEM analysis of TAM **9** localization. (**A**,**B**) Time-domain ESEEM signals for TAM **9** (**B**) with and (**A**) without liposomes (blue lines), with corresponding relaxation baselines (red lines). (**C**) Corresponding Fourier-transform (FT) spectra, normalized and baseline-corrected, showing the deuterium peak for each condition. (**D**) Comparison of FT spectra for TAM **9** and TAM **3** within liposomes. The higher deuterium peak intensity for TAM **9** and its concurrently lower hydrogen peak intensity (not shown explicitly but inferred) indicate that TAM **3** is embedded more deeply within the hydrophobic core of the liposomal membrane, limiting its access to solvent D_2_O.

This technique is particularly useful for probing the accessibility of water to a radical by comparing modulation amplitudes in deuterated solvent versus in liposomes. The ESEEM signal intensity is proportional to the concentration of unbound deuterium nuclei within a ~1 nm radius of the radical. When a radical is embedded within a lipid bilayer, the access of D_2_O molecules is hindered, leading to a reduced ESEEM signal compared to the radical in a homogeneous deuterated solution (Figure 7).

The experimental ESEEM spectra for TAM **9** and TAM **3** show two peaks at 2.22 MHz and 14.75 MHz, corresponding to the Larmor frequencies of deuterium and protons, respectively, at a magnetic field of ~350 mT. The intensities of these peaks are quantified in Table 3.

To accurately compare deuterium signal intensities, the different solvent conditions must be considered. TAM **9** was measured in a 50/50 D_2_O/glycerol mixture without lipids and in pure D_2_O with lipids. Since the deuterium nucleus density in the 50/50 mixture is approximately half that of pure D_2_O, the recorded intensities were weighted accordingly. After this correction, the deuterium signal for TAM **9** in liposomes was 5.5 times weaker than in the homogeneous solution. A similar reduction (a factor of 6) was observed for TAM **3**. This significant decrease in deuterium signal for both radicals confirms their embedding within the lipid membrane, which severely restricts water access to the radical centers.

A comparison of the Fourier-transformed ESEEM spectra for TAM **9** and TAM **3** in liposomes (Figure 7D) reveals a markedly lower deuterium peak intensity for TAM **3**, indicating it resides deeper within the lipid bilayer, where access to solvent D_2_O is more restricted. This conclusion is further supported by the concurrently higher hydrogen peak intensity for TAM **3**, which originates primarily from the proton-rich lipid acyl chains. While the radicals themselves contribute a small, consistent proton signal (see Supplementary Materials), the significant increase for TAM **3** confirms its deeper integration.

The shallower localization of TAM **9** relative to TAM **3** and the similarly embedded TAM **7** (see Supporting Information) is attributed to its polar carboxyl group. This hydrophilic substituent increases the molecule’s affinity for the membrane-water interface, favoring a position in the upper regions of the bilayer.

The work of Dzuba and co-workers demonstrated that solvent accessibility, as measured by ESEEM, is a reliable indicator of membrane depth [49]. They showed that water penetrates deeply into lipid bilayers, reaching the level of the tenth carbon atom in DPPC fatty acid chains. This implies that even membrane-embedded radicals can maintain significant water contact.

Our results confirm that TAM **3**, **7**, and **9** all localize within the lipid bilayer, where their mobility is restricted. However, their specific positions and hydration differ markedly. The nonpolar TAM **3** and **7** reside deep within the hydrophobic core, exhibiting minimal water accessibility. In contrast, the amphiphilic TAM **9**, with its polar carboxyl group, is anchored closer to the membrane-water interface, maintaining greater hydration. This superior water accessibility is expected to make TAM **9** a more effective polarizing agent for DNP applications.

## 3. Materials and Methods

### 3.1. Synthesis of TAM Radicals 3–9

Trityl radicals **1** [50], **3** [31], and **11** [34] were prepared according to previously described procedures. The syntheses of compounds **4**–**9** are described in the Supporting Information.

### 3.2. Sample Preparation for EPR Measurements

Samples of TAM **3–8** were prepared in toluene at a concentration of 10^−4^ M. Liposome samples containing TAM **3** or TAM **9** were prepared as described previously. All samples were deoxygenated using a “freeze–pump–thaw” method, with toluene solutions undergoing a minimum of three cycles and liposome samples undergoing five cycles. After each thawing step, the samples were purged with helium. Following deoxygenation, all samples were sealed in capillaries under reduced pressure.

### 3.3. CW-EPR Spectroscopy

X-band CW-EPR spectra were recorded at room temperature on a Bruker ELEXSYS E580 spectrometer (Karlsruhe, Germany) equipped with an ER4119HS high-sensitivity resonator. Samples were loaded into 50 μL Hirschmann Ringcaps capillaries. The acquisition parameters were as follows: sweep width, 3.5 mT; microwave power, 0.2 mW; modulation frequency, 100 kHz; modulation amplitude, 10 µT; sweep time, 120.01 s; conversion time, 58.60 ms; number of points, 2048; and 16 scans per spectrum.

### 3.4. Spectral Simulation

CW-EPR spectral simulations were performed using the EasySpin software package (version 6.0.7) in the fast-motion regime [51,52]. For TAM **3**–**8** in toluene, initial parameters (g-factor, linewidth, and anisotropic hyperfine constants) were taken from references [35,41]. Variable parameters for the simulation included the isotropic g-factor, Gaussian and Lorentzian linewidth components for the ^13^C_1_ center line, and the isotropic hyperfine constant with corresponding linewidth components for the satellite lines. The g-factors for the ^12^C_1_ and ^13^C_1_ spin systems were assumed to be identical.

Simulations of the CW-EPR spectra for TAM **3** and **9** with liposomes were also conducted in the fast-motion regime. Initial parameters were taken from the same literature, with the isotropic g-factor and Gaussian/Lorentzian linewidth components for the ^12^C_1_ center line as the variable parameters.

### 3.5. Pulse EPR Measurements and Analysis

Echo-detected EPR spectra, electron spin relaxation times, and ESEEM data were acquired on a homemade X-band pulsed EPR spectrometer [53]. All experiments were performed at 80 K using a π-pulse duration of 40 ns and a π/2-pulse duration of 20 ns. The transverse relaxation time T_m_ was determined with a standard Hahn echo sequence, while the longitudinal relaxation time T_1_ was measured using an inversion recovery (π—τ—π/2—T—π) sequence. A three-pulse sequence was employed for the ESEEM measurements.

### 3.6. Preparation of the Liposomes

Liposomes were prepared from DOPC lipid. The radicals were incorporated at a concentration of 0.25 mol% by first dissolving the components in chloroform. After solvent removal under reduced pressure, the sample was kept under vacuum for 4 h. The dried lipid film was then hydrated with a buffer solution and sonicated for 30 min. The final solution was centrifuged, resulting in the formation of a liposomal film at the surface. At elevated trityl concentrations, the solution became colored. The film was visibly more intense in color than the supernatant, confirming the successful incorporation of the trityl radicals into the liposomes during centrifugation.

## 4. Conclusions

In this work, we report the systematic synthesis and spectroscopic characterization of new lipophilic and amphiphilic Finland trityl derivatives, designed to modulate bilayer interactions through controlled variation in substituent properties. Continuous-wave and pulse EPR studies confirm that the narrow EPR linewidth of the trityl core—essential for efficient microwave saturation—is preserved across all derivatives. Crucially, ESEEM experiments in DOPC liposomes reveal that the amphiphilic TAM **9**, bearing a single cholesterol anchor and a polar carboxyl group, is optimally positioned in the bilayer: sufficiently anchored to minimize motional averaging yet accessible to solvent protons. This dual character creates ideal conditions for the Solid Effect DNP at ambient temperatures. In contrast, nonpolar derivatives (e.g., TAM **3** and **7**) embed deeply within the hydrophobic core, limiting water access and reducing their efficacy as polarizing agents. These findings establish TAM **9** as a prime candidate for DNP applications in membrane biophysics and provide a rational framework for designing next-generation polarizing agents for complex biological systems. Furthermore, the inherent hydrophilicity of the OX063 radical suggests its derivatives may also yield promising DNP performance, warranting future investigation.

## Figures and Tables

**Table 1:** HFI constants ^13^C for TAM radicals **3**–**8** derived from CW-EPR spectral simulations. The sequence numbers of ^13^C atoms are shown in Figure 5. RMSD value for each ^13^C HFI constant measurement was approximately ±0.02 mT, except for a_1_ with a value of ±0.05 mT.

	a_1_/mT	a_2_/mT	a_3_/mT	a_7_/mT	a_4,6_/mT	a_5_/mT	a_8_/mT
TAM **3**	2.37	1.1	0.88	0.88	0.29	0.21	0.06
TAM **4**	2.38	1.12	0.89	0.89	0.32	0.23	0.05
TAM **5**	2.35	1.1	0.87	0.87	0.31	0.22	0.06
TAM **6**	2.35	1.11	0.82	0.97	0.36	0.24	0.04
TAM **7**	2.36	1.1	0.88	0.88	0.29	0.21	0.05
TAM **8**	2.38	1.1	0.88	0.88	0.3	0.21	0.06

**Table 2:** Magnetic resonance parameters for TAM radicals **3**–**8** in toluene and for TAM **3** and **9** with liposomes at room temperature, derived from X-band CW-EPR spectral simulations. Listed are the Gaussian and Lorentzian contributions to the full width at half maximum (FWHM, in mT) of the central ^12^C_1_ line. The Voigt linewidth profile was estimated using established approximations [36,37]. RMSD value for each linewidth measurement was approximately ±0.01 mT.

Compound	Line Width (Gaussian)/mT	Line Width (Lorentzian)/mT	Line Width (Voight)/mT
TAM **3**	0.042	0.035	0.064
TAM **4**	0.059	0.030	0.077
TAM **5**	0.041	0.035	0.063
TAM **6**	0.043	0.023	0.057
TAM **7**	0.038	0.002	0.039
TAM **8**	0.024	0.004	0.026
TAM **3** *^,1^	0.043	0.030	0.061
TAM **3** *^,2^	0.117	0.192	0.250
TAM **9** *	0.003	0.116	0.116

* CW-EPR magnetic resonance parameters for compounds were acquired with liposomes. ^1^ CW-EPR spectral parameters for narrow singlet EPR signal component. ^2^ CW-EPR spectral parameters for wide singlet EPR signal component.

**Table 3:** Deuterium (2.22 MHz) and proton (14.75 MHz) peak characteristics from the Fourier-transform ESEEM spectra of TAM **3** and TAM **9**, with and without liposomes. Values for both the intensity (amplitude) and integrated area of each peak are provided.

	D		H	
	Max	Intensity	Max	Intensity
TAM **9** ^1^	1.00	7.7 ± 0.5	0.45 ± 0.05	3.2 ± 0.5
TAM **9** ^2^	0.40 ± 0.05	2.8 ± 0.5	0.56 ± 0.05	3.8 ± 0.5
TAM **3** ^1^	1.00	6.3 ± 0.5	0.10 ± 0.05	0.70
TAM **3** ^2^	0.08 ± 0.05	0.7 ± 0.5	0.19 ± 0.05	1.37

^1^ EPR spectral parameters for samples without liposomes. ^2^ EPR spectral parameters for samples with liposomes.

## Data Availability

No datasets were generated or analyzed during the current study.

## Supplementary Materials

The following supporting information can be downloaded at https://www.mdpi.com/article/10.3390/molecules30224463/s1. Descriptions of general procedures; Descriptions of TAM **3**–**9** syntheses; Scheme S1: Syntheses of TAMs **5** and **6**; Scheme S2: Syntheses of TAMs **7**, **8** and **9**; Figure S1: Fourier spectra of TAM **3** in liposomes and in deuteromethanol; Figure S2: Fourier spectra of TAM **3** and TAM **7** in liposomes and in deuterated water; Figure S3: Echo-detected spectra of TAM **3**–**8** with time dependences of the electron spin echo signal intensities; Figure S4: Echo detected EPR spectra of TAM **9** with liposomes and D_2_O without liposomes in D_2_O/glycerol mixture; Figure S5: The kinetics of the electron spin echo decay obtained in the three-pulse ESEEM experiment for TAM **9** with liposomes and D_2_O (T_1_ measurements); Figure S6: The kinetics of the electron spin echo decay obtained in the three-pulse ESEEM experiment for TAM **9** without liposomes in D_2_O/glycerol mixture (T_1_ measurements); Figure S7: The kinetics of the electron spin echo decay for TAM **3**–**9**; Figure S8: The kinetics of the electron spin echo decay obtained in the three-pulse ESEEM experiment for TAM **9** with liposomes and D_2_O (T_m_ measurements); Figure S9: The kinetics of the electron spin echo decay obtained in the three-pulse ESEEM experiment for TAM **9** without liposomes in D_2_O/glycerol mixture (T_m_ measurements). Table S1 (The electron spin relaxation times T_m_, T_1_ of TAM 3-8 solutions in toluene). The authors cite additional references within the Supporting Information [54,55].

## Abbreviations

The following abbreviations are used in this manuscript:

TAM	Triarylmethyl radicals
DNP	Dynamic nuclear polarization
SE DNP	Solid-effect DNP mechanism
ESEEM	Electron spin echo envelope modulations
HFI	Hyperfine interaction
DOPC	1,2-dioleoyl-sn-glycero-3-phosphocholine
BDPA	1,3-bis(diphenylene)-2-phenylallyl
DIPEA	Diisopropylethylamine
HATU	Hexafluorophosphate Azabenzotriazole Tetramethyl Uronium
