# Supplementary material for: Paramagnetic Agents for SE DNP: Synthesis and ESR Characterization of New Lipophilic Derivatives of Finland Trityl
Source: Molecules. 2025 Nov 19;30(22):4463. doi: 10.3390/molecules30224463 (PMC12655640; doi:10.3390/molecules30224463)
Supplement: Supplementary file 1 [file molecules-30-04463-s001.zip › molecules-3992293-supplementary.pdf]

Supplementary Materials

Paramagnetic Agents for SE DNP: Synthesis and ESR Characterization of New Lipophilic Derivatives of Finland trityl

Victor M. Tormyshev[1,*], Danil A. Kuznetsov[1,2], Arthur E. Raizvikh[1,2], Olga Yu. Rogozhnikova[1], Tatiana I. Troitskaya[1], and Elena G. Bagrynskaya[1,*]

[1] *N.N. Vorozhtsov Novosibirsk Institute of Organic Chemistry, Siberian Branch of the Russian Academy of the Sciences (SB RAS), Novosibirsk 630090, Russia*; torm@nioch.nsc.ru (V.M.T.); d.kuznetsov1@g.nsu.ru (D.A.K.); arturaiz@nioch.nsc.ru (A.E.R.); rogol@nioch.nsc.ru (O.Y.R.); egbagryanskaya@nioch.nsc.ru (E.G.B.)

[2] *Physical Department, Novosibirsk State University, Novosibirsk 630090, Russia*

* Correspondence: torm@nioch.nsc.ru, egbagryanskaya@nioch.nsc.ru

Table of contents

1 Experimental. S2

1.1 General Procedures. S2

1.2 Synthesis of TAM radicals **3-9**. S2

1.2.1 Scheme S1. Syntheses of TAMs **5** and **6**. S3

1.2.2 Scheme S2. Syntheses of TAMs **7**, **8** and **9**. S4

2 ESEEM measurements. S5

2.1 Figure S1. Fourier transform of the ESEEM of TAM **3** in liposomes and in deuteromethanol. S5

2.2 Figure S2. Fourier transform of the ESEEM of TAM **3** and TAM **7** in liposomes and in deuterated water. S6

2.3 Figure S3. Echo-detected spectra of TAM **3-8** with time dependences of the electron spin echo signal intensities. S6

2.4 Figure S4. Echo detected EPR spectra of TAM **9** with liposomes and D_2O without liposomes in D_2O/glycerol mixture. S6

2.5 Figure S5. The kinetics of the electron spin echo decay obtained in the three-pulse ESEEM experiment for TAM **9** with liposomes and D_2O (T_1 measurements). S7

2.6 Figure S6. The kinetics of the electron spin echo decay obtained in the three-pulse ESEEM experiment for TAM **9** without liposomes in D_2O/glycerol mixture (T_1 measurements). S7
2.7 Figure S7. The kinetics of the electron spin echo decay for TAM **3-9**. S8
2.8 Figure S8. The kinetics of the electron spin echo decay obtained in the three-pulse ESEEM experiment for TAM **9** with liposomes and D_2O (T_m measurements). S8
2.9 Figure S9. The kinetics of the electron spin echo decay obtained in the three-pulse ESEEM experiment for TAM **9** without liposomes in D_2O/glycerol mixture (T_m measurements). S9
2.10 Table S1. The electron spin relaxation times Tm, T1 of TAM 3-8 in toluene. S9
3 References. S10

Experimental

General Procedures

^{1}H and ^{13}C NMR spectra were recorded on a Bruker AV-400 spectrometer (^{1}H: 400 MHz, ^{13}C: 100 MHz). Chemical shifts (δ scale) are given in ppm with reference to residual signals of chloroform-D (^{1}H: 7.26, ^{13}C: 77.16). IR spectra were recorded on a Bruker Vector 22 FTIR spectrometers in KBr pellets, wavenumber values are given in cm^{-1}. The melting points were measured with a Boetius hot stage and are uncorrected. Preparative column chromatography was performed using 60–200 μm silica gel purchased from Acros. Chemicals were purchased from Aldrich and Acros and were used without further purification. The solvents of pure and ultrapure grade were purified according to standard procedures followed by distillation.

For treatment of samples with ultrasound the Sonorex Super RK 103 H Ultrasound Bath was used (BANDELIN electronic GmbH & Co. KG, ultrasound frequency 35kHz, ultrasound power 140 W).

Synthesis of TAM radicals 3-9

Trityl radicals **1** [1]**, 3** [2] and **11** [3] were obtained according to previously described procedures.

TAM **4** (Scheme 1). A 10-mL pear-shaped one-necked flask equipped with magnetic stirring bar was charged with TAM **1** (42.4. mg, 0.042 mmol), HATU (58 mg, 0.153 mmol), n-octadecylamine (42 mg, 0.158 mmol) and solution of DIPEA (16 mg, 0.127 mmol) in anhydrous DMF (0.5 mL). The resulting reaction mixture was treated with ultrasound and stirred at room temperature overnight under argon. The mixture obtained after addition of water (5 mL) and 0.1 M solution of $NaHSO_4$ (1.3 mL) was extracted with DCM (5x3 mL). The combined organic extract was filtered through a short plug of silica gel and concentrated in vacuo. Column chromatography on silica gel with DCM followed by DCM/methanol (100:1 v/v) used as eluents afforded TAM **4** (0. 042 g, 57 %). Black powder, m.p. > 200 ^oC (with decomposition). Found (%): C, 64.12; H, 8.54; N, 2.38. $C_{94}H_{150}N_3O_3S_{12}$ requires (%): C, 64.33; H, 8.62; N, 2.39. **IR** (KBr): $\tilde{\nu}$ = 2922 (vs), 2852 (s), 1647 (s), 1520 (s), 1452 (m), 1435 (m), 1365 (m), 1298 (m), 1240 (s), 1149 (m). **EPR** (0.1 mM in DCM solution: broad singlet, linewidth 196 mGs.

Amine **10** (Scheme S1). To a stirred pale-yellow solution of 7,11,18,21-tetraoxa-3,15-diazatrispiro[5.2.2.5.2.2]heneicosane dihydrochloride (**13** [4], 1.11g , 3 mmol) and triethylamine (1.01 g, 10 mmol) in methanol (25 mL) slowly over 40 min was added a solution of di-tert-butyl dicarbonate (0.655 g, 3 mmol) in THF (2 mL). The resulting reaction mixture was stirred at room temperature overnight after which it was concentrated in vacuo to dryness. The crystalline cake was suspended THF (15 mL). A solution of KOH (0.336 g, 6 mmol) in water (0.5 mL) and methanol (0.5 mL) was added. The resulting turbid mixture was left standing for 30 min and then filtered through paper. Solvents were removed in vacuo. The resulting pale-yellow caramel was dissolved in DCM (10 mL) to give a slightly turbid solution it was filtered and concentrated in vacuo. Column chromatography on silica gel with DCM followed by methanol and methanol/aqueous ammonia (100:1 v/v) used as eluents gave amine **10** as a pale-yellow powder (0. 536 g, 45 %). Found (%): C, 60.42; H, 8.64; N, 6.96. $C_{20}H_{34}N_2O_6$ requires (%): C, 60.28; H, 8.60; N, 7.03. ^{1}H NMR (400 MHz, $CDCl_3$): 1.44 (s, 9H), 1.50 (broad s, 1H), 1.78 (multiplet, 8H), 2.84 (multiplet, 4H), 3.42 (multiplet, 4H), 3.74 (s, 8H). ^{13}C NMR (100 MHz, $CDCl_3$): 28.58, 32.26, 32.28, 33.26, 33.93, 63.32, 63.66, 79.72, 97.29, 97.55, 154.88.

TAM **5** (Scheme S1).). A 10-mL pear-shaped flask equipped with magnetic stirring bar was charged with TAM **11** (50.8. mg, 0.0494 mmol), HATU (22.5 mg, 0.0593 mmol), amine **10** (25.6 mg, 0.0643 mmol) and solution of DIPEA (6.4 mg, 0.0494 mmol) in anhydrous DMF (0.5 mL). The resulting reaction mixture was treated with ultrasound and stirred at room temperature overnight under argon. The mixture obtained after addition of water (5 mL) and 0.1 M solution of $NaHSO_4$ (2 mL) was extracted with DCM (5x3 mL). The combined organic extract was filtered through a short plug of silica gel and concentrated in vacuo. Column chromatography on silica gel with DCM followed by DCM/methanol (50:1 v/v) used as eluents afforded TAM **5** (0. 059 g, 84 %). Black powder, m.p. > 220 ^{o}C (with decomposition). Found (%): C, 52.91; H, 5.29; N, 2.05. $C_{62}H_{75}N_2O_{11}S_{12}$ requires (%): C, 52.85; H, 5.37; N, 1.99. **IR** (KBr): $\tilde{\nu}$ = 2956 (m), 2924 (m), 2858 (m), 1702 (s), 1643 (m), 1450 (m), 1435 (m), 1383 (m), 1365 (m), 1267 (m), 1238 (vs), 1169 (m), 1147 (m), 1138 (m), 1093 (s). **EPR** (0.1 mM in DCM solution: broad singlet, linewidth 179 mGs.

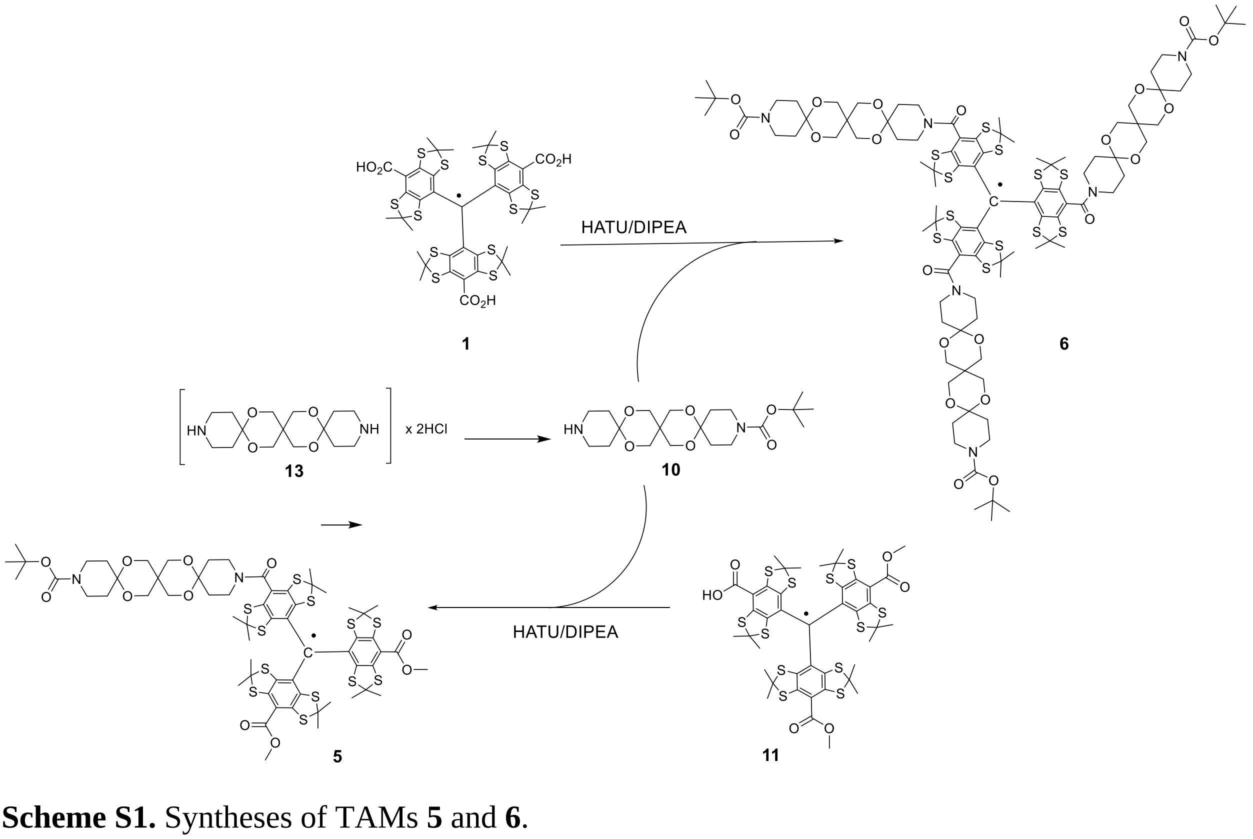

Scheme S1. Syntheses of TAMs **5** and **6**.

TAM **6**. TAM **6** was obtained by the method described above for the synthesis of TAM **4.** Black powder, m.p. > 220 °C (with decomposition). Yield: 61 %. Found (%): C, 55.96; H, 6.35; N, 3.90. $C_{100}H_{135}N_6O_{21}S_{12}$ requires (%): C, 56.08; H, 6.35; N, 3.92. **IR** (KBr): $\tilde{\nu}$ = 2964 (m), 2928 (m), 2866 (m), 1695 (s), 1643 (s), 1466 (m), 1429 (m), 1383 (m), 1365 (m), 1265 (m), 1240 (s), 1169 (s), 1142 (m), 1093 (vs). **EPR** (0.1 mM in DCM solution: broad singlet, linewidth 120 mGs.

Ester **12**. Bromoacetate **12** (Scheme S2) was obtained using the known literature method [5]. ^{1}H NMR (400 MHz, $CDCl_3$): 0.67 (s, 3H), 0.85 (d, 3H, *J* 1.8 Hz), 0.86 (d, 3H, *J* 1.8 Hz), 0.91 (d, 3H, *J* 6.5 Hz), 1.02 (s, 3H), 2.35 (d, 2H, *J* 7.7 Hz), 3.82 (s, 2H), 4.66 (m, 1H) 5.39 (d, 1H, *J* 4.1 Hz). ^{13}C NMR (100 MHz, $CDCl_3$): 11.97, 18.82, 19.43, 21.12, 22.71, 22.98, 23.93, 24.39, 26.61, 27.60, 28.14, 28.36, 31.91, 32.00, 35.91, 36.27, 36,65, 36.95, 37.87, 39.62, 39.78, 42.39, 50.03, 56.17, 56.74, 76.25, 123.27, 139.21, 166.82.

TAM **7**. To a stirred solution of TAM **11** (48.5 mg, 0.047 mmol) and DIPEA (12.2 mg, 0.094 mmol) in anhydrous THF (0.5 mL) was added a solution of ester **12** (36.0 mg, 0.071 mmol) in THF (0.5 mL). The resulting reaction mixture was stirred at 45 ^{0}C for 36 h, after which the reaction was quenched by addition of water (2.5 mL) and 2M HCl (0.12 mL). The mixture was extracted with DCM (5x3 mL). The combined organic extract was filtered through a short plug of silica gel and concentrated in vacuo. Column chromatography on silica gel with DCM afforded TAM **7** (57.5 mg, 84%).Black powder, m.p. > 200 °C (with decomposition). Found (%): C, 58.51; H, 6.18; O, 8.74. $C_{71}H_{89}O_8S_{12}$ requires (%): C, 58.60; H, 6.16; O, 8.80. **IR** (KBr): $\tilde{\nu}$ = 2951 (m), 2928 (m), 2866 (m), 1709 (s), 1491 (m), 1454 (m), 1435 (m), 1383 (m), 1365 (m), 1319 m), 1306 (m), 1275 (m), 1234 (vs), 1200 (m), 1167 (m), 1147 (m), 1135 (m), 1113 (m). **EPR** (0.1 mM in DCM solution: broad singlet, linewidth 135 mGs.

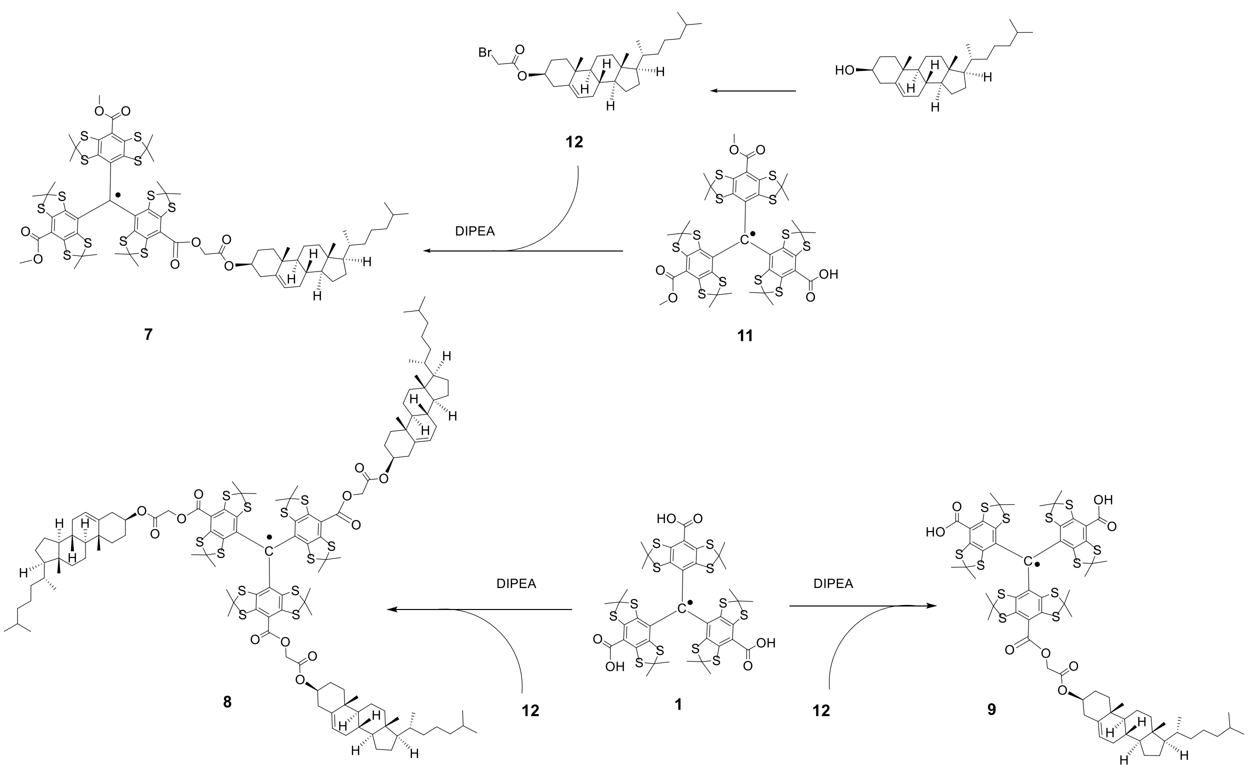

Scheme S2. Syntheses of TAMs **7**, **8** and **9**.

TAM **8**. A solution of TAM **1** (43.4 mg, 0.0433 mmol), DIPEA (22.4 mg, 0.173 mmol), and ester **12** (87.9 mg, 0.173 mmol) in anhydrous THF (1.2 mL) was stirred at 45 ^{0}C for 36 h. The reaction was quenched by addition of water (2.5 mL) and 2M HCl (0.12 mL). The mixture was extracted with DCM (5x3 mL). The combined organic extract was filtered through a short plug

of silica gel and concentrated in vacuo. Column chromatography on silica gel with DCM/hexane (3:1, v/v) followed by DCM afforded TAM **8** (75.0 mg, 76%). Black powder, m.p. > 200 ^{o}C (with decomposition). Found (%): C, 66.94; H, 7.85; O, 8.50. $C_{127}H_{177}O_{12}S_{12}$ requires (%): C, 66.89; H, 7.82; O, 8.42. **IR** (KBr): $\tilde{\nu}$ = 2951 (vs), 2866 (s), 2852(s), 1765 (m), 1742 (s), 1711 (s), 1489 (m), 1468 (m), 1454 (m), 1435 (m), 1381 (m), 1290 (s), 1232 (vs), 1197 (vs), 1169 (m), 1134 (s), 1111 (s), 1055 (m), 1028 (m), 1007 (m), 997 (m). **EPR** (0.1 mM in DCM solution: broad singlet, linewidth 130 mGs.

TAM **9**. A solution of TAM **1** (51.7 mg, 0.0516 mmol), DIPEA (13.3 mg, 0.103 mmol), and ester **12** (31.4 mg, 0.062 mmol) in anhydrous THF (1.0 mL) was stirred at 45 ^{0}C for 48 h. The reaction was quenched by addition of water (2.5 mL) and 2M HCl (0.10 mL). The mixture was extracted with DCM (5x5 mL). The combined organic extract was filtered through a short cotton plug and concentrated in vacuo. Column chromatography on silica gel using DCM/methanol mixtures (with DCM/Mmethanol ratio gradually changed from 10:1 to 1:1, v/v) afforded TAM **9** (20.0 mg, 27%). Black powder, m.p. > 220 ^{o}C (with decomposition). Found (%): C, 57.96; H, 6.02; O, 8.92. $C_{69}H_{85}O_{8}S_{12}$ requires (%): C, 58.07; H, 6.00; O, 8.97. **IR** (KBr): $\tilde{\nu}$ = 2953 (s), 2955 (s), 2864 (m), 2854 (m), 1765 (m), 1737 ms), 1711 (s), 1581 (s), 1491 (m), 1454 (m), 1435 (m), 1382 (s), 1365 (s), 1317 (m), 1302 (m), 1234 vs), 1199 (m), 1167 (m), 1147 (m), 1133 (m), 1113 (m). **EPR** (0.1 mM in DCM/methanol (3:1) solution: broad singlet, linewidth 175 mGs.

ESEEM measurements

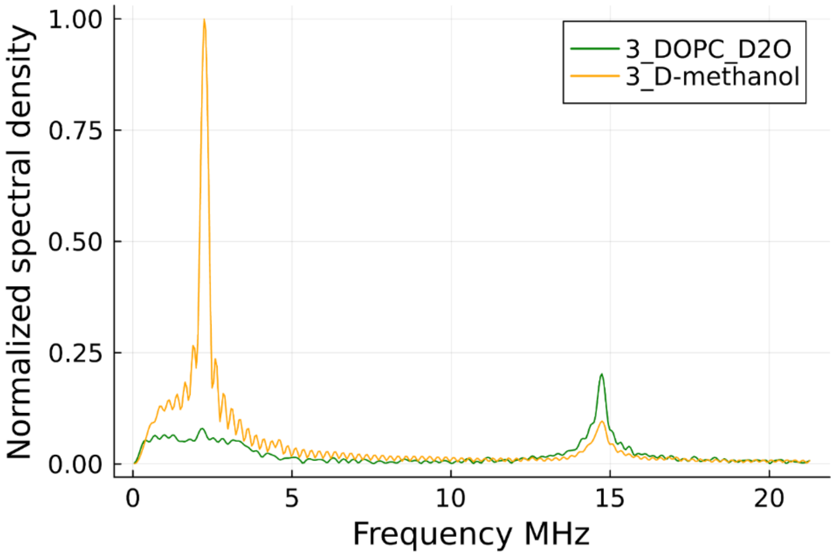

Figure S1 Fourier transform of the ESEEM of TAM **3** in liposomes (green) and in CD_3OD (orange). The concentration of deuterium nuclei in CD_3OD is ~1.6 times higher than in D_2O. Then the weighted ratio of the deuterium peak intensities is ~39.8/4.2/1.6 = 6.

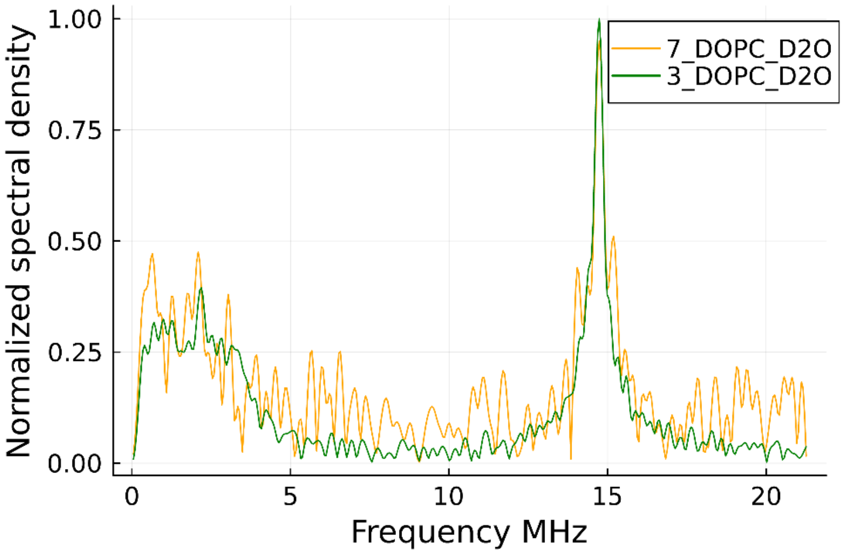

Figure S2. Fourier transform of the ESEEM of TAM **3** (green) and TAM **7** (orange) in liposomes and in deuterated water.

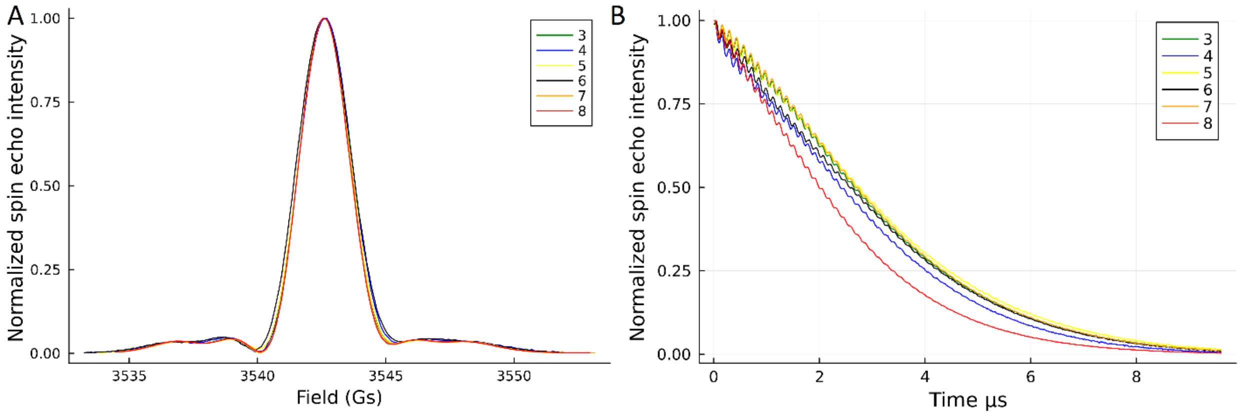

Figure S3. A) Echo-detected (ED) spectra of TAM **3-8.** B) Time dependences of the intensity of the electron spin echo signal of TAM **3-8** in 2-pulse echo decay at a temperature of 80 K. The samples were prepared in a toluene solution at a concentration of 10^{-4} mol/l**.**

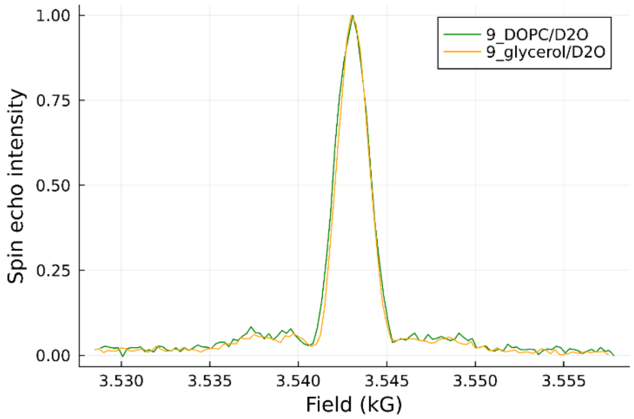

Figure S4. Echo detected (ED) spectra of TAM **9** with liposomes and D_2O (green) and without liposomes in D_2O/glycerol mixture (orange) at a temperature of 80 K. The spectrum was obtained using a two-pulse sequence π/2 - 20 ns π - 40 ns.

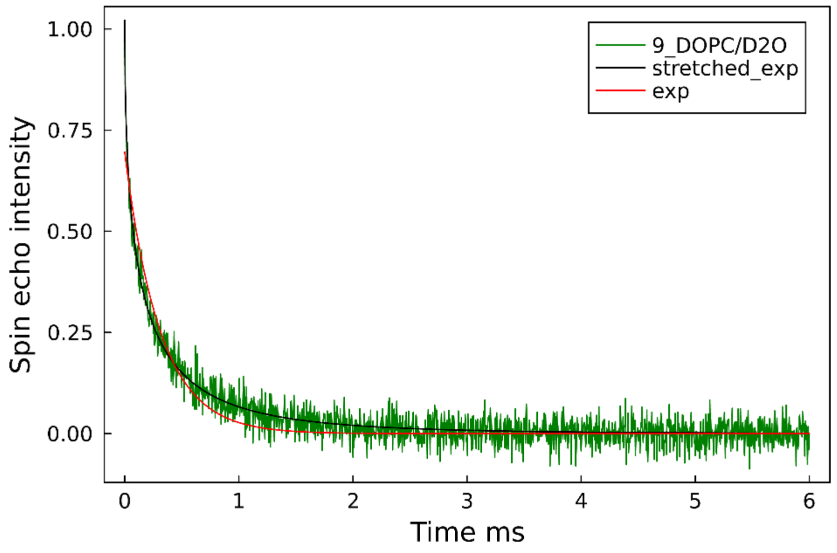

Figure S5. The kinetics of the electron spin echo decay obtained in the three-pulse ESEEM experiment for TAM **9** with liposomes and D_2O (green). Pulse length of π/2 was 20 ns at a temperature of 80 K. Solid lines represent simulations by mono-exponent (red) with T_1 = 0.3 ms and stretched exponent $e^{-(\frac{t}{T_1})^{\beta}}$ (black) with T_1 = 0.14 ms, β = 0.52.

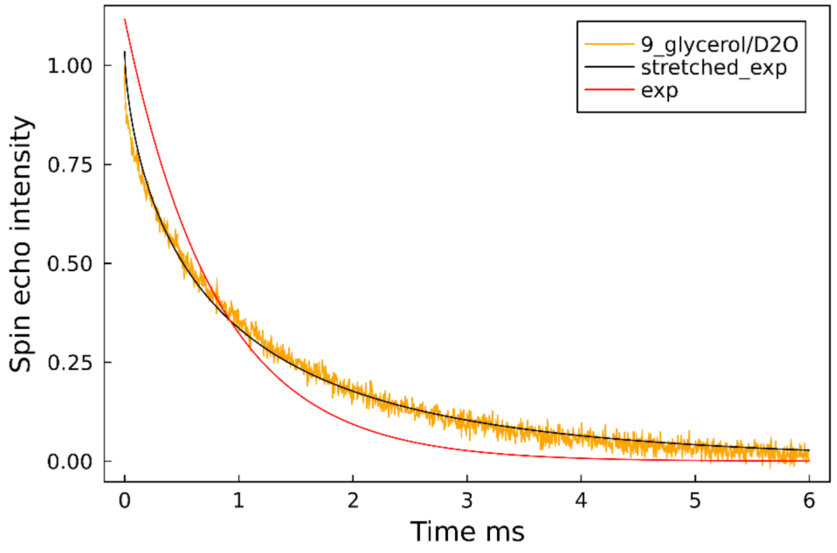

Figure S6. The kinetics of the electron spin echo decay obtained in the three-pulse ESEEM experiment for TAM **9** without liposomes in D_2O/glycerol mixture (orange) with a concentration of 10^{-4} mol/l at a temperature of 80 K. Pulse length of π/2 was 20 ns. Solid lines represent simulations by mono-exponent (red) with T_1 = 0.8 ms and stretched exponent $e^{-(\frac{t}{T_1})^{\beta}}$ (black) with T_1 = 0.84 ms, β = 0.65.

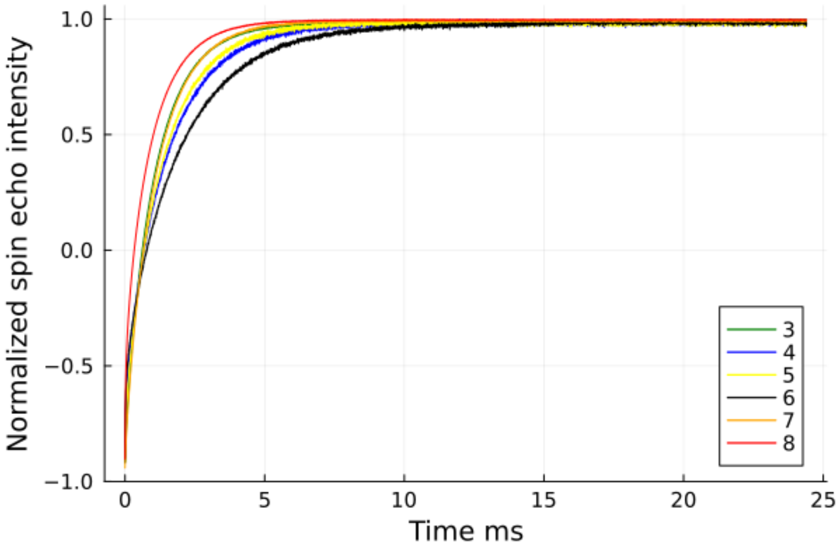

Figure S7. The kinetics of the electron spin echo decay for TAM **3-9** in a toluene solution at a concentration of 10^{-4} mol/l obtained using a π -τ - π/2 - T - π inversion recovery sequence π/2 - 20 ns π - 40 ns at a temperature of 80 K.

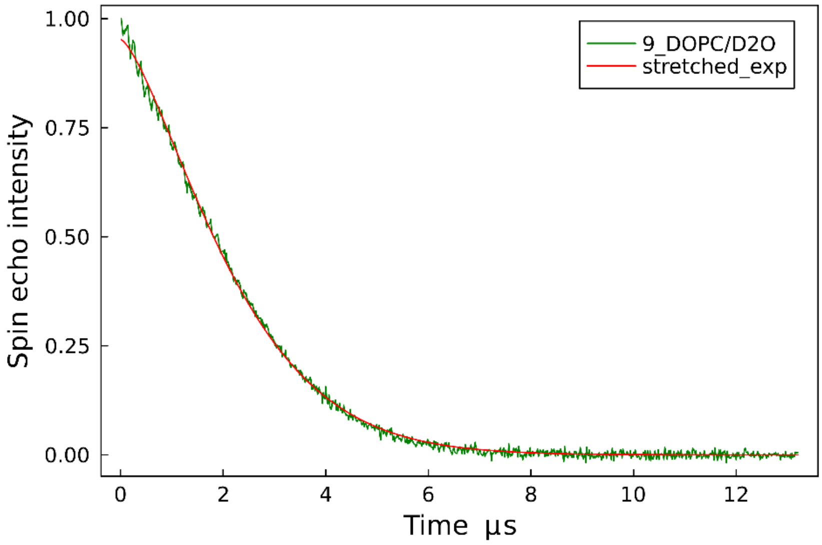

Figure S8. The kinetics of the electron spin echo decay for TAM **9** using a two-pulse sequence π/2 - 20 ns π - 40 ns with liposomes and D_2O at a temperature of 80 K. Solid line represents simulation by a stretched exponent $e^{-(\frac{t}{Tm})^{\beta}}$ (black) with T_m = 2.5 µs, β = 1.42.

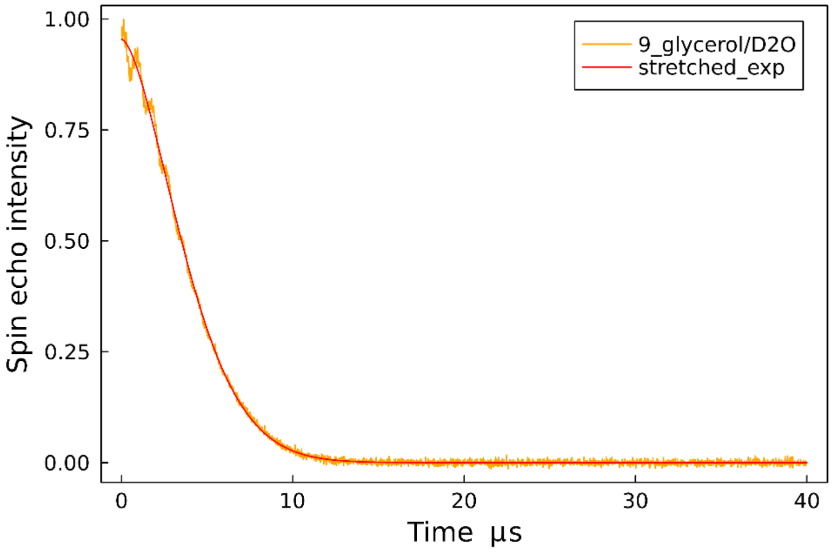

Figure S9. The kinetics of the electron spin echo decay for TAM **9** using a two-pulse sequence π/2 - 20 ns π - 40 ns without liposomes in D_2O/glycerol mixture at a temperature of 80 K. Solid line represents simulation by a stretched exponent $e^{-(\frac{t}{Tm})^{\beta}}$ (black) with T_m = 4.6 μs, β = 1.63.

Table S1. The electron spin relaxation times T_m , T_1 of TAM 3-8 solutions in toluene with a concentration of 10^{-4} mol/l at a temperature of 80 K. T_m is the time of transverse electron relaxation, T_1 is the time of longitudinal electronic relaxation, β is the coefficient of the stretched exponent $e^{-(\frac{t}{Tm})^{\beta}}$.

Radical	T_m μs ± 0.10 μs	β	T_1 ms ± 0.15 ms
TAM **3**	3.51	1.47	1.00
TAM **4**	3.34	1.46	1.51
TAM **5**	3.62	1.46	1.40
TAM **6**	3.47	1.41	1.87
TAM **7**	3.55	1.49	1.03
TAM **8**	2.71	1.36	0.85

References

1. Rogozhnikova, O.Y.; Vasiliev, V.G.; Troitskaya, T.I.; Trukhin, D.V.; Mikhalina, T.V.; Halpern, H.J.; Tormyshev, V.M. Generation of Trityl Radicals by Nucleophilic Quenching of Tris (2, 3, 5, 6-tetrathiaaryl) methyl Cations and Practical and Convenient Large-Scale Synthesis of Persistent Tris (4-carboxy-2, 3, 5, 6-tetrathiaaryl) methyl Radical. *European journal of organic chemistry* **2013**, *2013*, 3347–3355.
2. Edeleva, M.V.; Marque, S.R.A.; Rogozhnikova, O.Y.; Tormyshev, V.M.; Troitskaya, T.I.; Bagryanskaya, E.G. Radical polymerization of radical-labeled monomers: The triarylmethyl-based radical monomer as an example. *Journal of Polymer Science Part A: Polymer Chemistry* **2018**, *56*, 2656–2664.
3. Trukhin, D.V.; Rogozhnikova, O.Y.; Troitskaya, T.I.; Vasiliev, V.G.; Bowman, M.K.; Tormyshev, V.M. Facile and high-yielding synthesis of TAM Biradicals and Monofunctional TAM radicals. *Synlett* **2016**, *27*, 893–899.
4. Moldovan, O.; Lameiras, P.; Nagy, I.; Opruta, T.; Popa, F.; Antheaume, C.; Ramondenc, Y.; Darabantu, M. Stereochemistry of six-membered spiranes arising from the first use of a diaza-trispiro-heneicosane motif in the synthesis of a G-1 dendritic melamine. *Tetrahedron* **2013**, *69*, 2199–2213.
5. Elenkov, I.J.; Todorova, D.I.; Bankova, V.S.; Milkova, T.S. Synthesis of steryl esters of phenolic acids by a heterogeneous Wittig reaction. *Journal of natural products* **1995**, *58*, 280–283.
